# A novel glycoform of the rotavirus outer capsid protein VP7 is secreted from polarized cells and activates innate immune cells

**DOI:** 10.1128/jvi.01898-25

**Published:** 2026-02-24

**Authors:** Ashley Nutsford, The Huong Chau, Carol Wang, Ash Sargent, Camren Cullen, Thomas Reilly, Morten Thaysen-Andersen, Anna E. S. Brooks, John A. Taylor

**Affiliations:** 1 School of Biological Sciences, University of Auckland 1415, Auckland, New Zealand; 2 School of Natural Sciences, Macquarie University 7788, North Ryde, Australia; 3 Nagoya University, Institute for Glyco-core Research 12965, Nagoya, Aichi, Japan; 4 Grafton Campus, University of Auckland, Liggins Institute 1415, Auckland, New Zealand; University of Michigan Medical School, Ann Arbor, Michigan, USA

**Keywords:** glycoprotein, glycomics, VP7, rotavirus

## Abstract

Rotaviruses (RVs) are unique among non-enveloped animal viruses encoding a structural and a nonstructural glycoprotein in their genome. The outer capsid of the virion is formed by 260 trimers of VP7, which is glycosylated at an asparagine residue(s) in the endoplasmic reticulum of infected cells, where it is retained until a late stage of virion morphogenesis. We investigated the fate of VP7 produced during synchronous infection of polarized Caco-2 cells and observed that a previously undetected form with a molecular weight greater than virion-associated VP7 was secreted into the medium. This secreted form of VP7 (sVP7) was not associated with virions and was insensitive to endoglycosidase H treatment consistent with the dominance of complex type N-glycans revealed in structural details using LC-MS/MS. We also detected core 1 and 2-type O-glycans on sVP7 contributing to the higher-than-predicted molecular weight. Purification of sVP7 from the medium of virus-infected cells revealed that it was trimeric and retained conformationally sensitive epitopes recognized by neutralizing monoclonal antibodies. Like the secreted nonstructural protein 4(NSP4), sVP7 exhibited agonism of TLR2 and TLR4 and caused activation of innate immune cells in human blood. Our results identify a novel form of the rotavirus capsid glycoprotein previously undetected and suggest that VP7 may possess extracellular functions beyond its role in the virion as a modulator of innate and adaptive antiviral immunity.

**IMPORTANCE:**

Rotavirus (RV) exhibits a mode of assembly unique among non-enveloped animal viruses whereby virion morphogenesis occurs within an endomembrane compartment. The outer shell of rotavirus is assembled within the lumen of the ER where a glycoprotein VP7 is transferred to immature particles that bud from cytoplasmic inclusions. Lytic release of virions from non-polarized cells has obscured the fate of rotavirus glycoproteins from cells with a differentiated phenotype. This study reveals that in polarized cells, a significant proportion of VP7 is secreted, independently of virions, and acquires novel glycan signatures through extensive modification of N- and O-linked glycosylation within the medial Golgi complex. Secreted VP7 exhibited agonism of Toll-like receptors and activated several distinct subsets of innate immune cells while retaining conformationally dependent neutralizing epitopes associated with a trimeric structure indicating a potential role for the secreted capsid glycoprotein in modulating both innate and adaptive immune responses to rotavirus infection.

## INTRODUCTION

Rotaviruses (RVs) belong to the family of non-enveloped *Sedo*r*eoviridae,* members of which have segmented dsRNA genomes (1). Mammalian rotaviruses infect a wide range of host species transmitted via the fecal-oral route and cause febrile gastroenteritis and diarrhea (2, 3). The virion is assembled from six structural proteins that form a non-enveloped, triple-layered particle (TLP) exhibiting T = 13 icosahedral symmetry (4). A further six non-structural proteins (NSPs) are synthesized in RV-infected cells and participate in virion morphogenesis or modulate the innate immune response to infection (3, 5).

Immortalized monkey kidney cells, primarily MA104, have been most frequently employed in studies of RV replication (6, 7). In RV-infected MA104 cells, viroplasms are evident in the cytoplasm by electron microscopy (8). Within viroplasms, immature double-layered particles (DLPs) are assembled and transferred into the lumen of the endoplasmic reticulum (ER) or an ER-derived compartment by a budding mechanism (9, 10). This process is initiated by interaction of the DLP with the non-structural glycoprotein NSP4 and results in the assembly of the outer capsid protein VP7 and the virion spike protein VP4 within the ER lumen. Virus release occurs by cell lysis in non-polarized MA104 cells. However, both the kinetics of virus production and the attendant loss of cell viability follow a more progressive timeline in polarized human colon adenocarcinoma (Caco-2) cells in which virus release can occur via a vesicular transport mechanism (11, 12)

A unique aspect of rotavirus replication is the maturation of virions within the ER requiring the translocation of VP7 across the ER membrane prior to assembly of the outer capsid layer. Expression of VP7 in cell lines implicated a cleavable N-terminal signal sequence and a further region of the polypeptide responsible for its ER retention in the absence of an apparent transmembrane domain (13, 14). The single site for N-linked glycans present on VP7 (Asn69) is considered to be decorated with oligomannosidic-type N-glycans, suggesting that the protein remains in the ER and does not enter the Golgi complex where further processing to complex-type N-glycans is known to occur (15).

Previously, we reported that the N-linked oligosaccharides attached to NSP4 were modified during its secretion from infected Caco-2 cells (16). Modification of glycan residues in secreted, but not cell-associated, forms of NSP4 indicated that rotavirus glycoproteins previously considered to be resident in endomembrane compartments in nonpolarized cells could have alternative fates in polarized cells. We have now extended these studies to VP7 during infection of polarized cells infected with RV. We report here that VP7 is also actively secreted from RV-infected Caco-2 cells independently of its association with virions or other viral proteins. sVP7 is trimeric and bound by conformation-dependent neutralizing antibodies while the secreted protein exhibited agonism of Toll-Like Receptors (TLRs) and activated several populations of innate immune cells. These observations hint at additional role(s) for VP7 beyond formation of the outer capsid layer of the virion, potentially as an antigen decoy or agonist of innate immune cells capable of influencing virus host interactions.

## MATERIALS AND METHODS

### Cell culture and virus infection

The cell culture-adapted simian rotavirus strain, SA11 (G3P[2]) was propagated in MA104 cells according to the protocol described by reference 6. Virus titer was measured in MA104 cells, using a fluorescent focus assay (FFA) as outlined previously (6). MA104 (ATCC: CRL-2378.1) and Caco-2 (ATCC: HTB37) cells were cultured in Dulbecco’s modified Eagle’s medium (DMEM, Gibco) supplemented with 10% fetal bovine serum (FBS, Moregate Biotech) and 100 µg/mL penicillin-streptomycin (Sigma). HEK-Blue cells (-null, -TLR2, -TLR4) were maintained in DMEM supplemented with heat-inactivated FBS, 100 mg/L Normocin (InvivoGen) with HEKBlue selection (InvivoGen) or Zeocin (InvivoGen). Cells were maintained with 5% CO_2_ at 37°C in a humidified incubator. Before infection, MA104 cells were washed twice with serum-free DMEM, while Caco-2 cells were maintained in serum-free DMEM for 6 h before addition of virus. For infection, the viral inoculum was activated with 1 µg/mL trypsin (Thermo Fisher Scientific) at 37°C for 60 min, then added to cells at the indicated multiplicity of infection (MOI) for 60 min at 37°C in 5% CO_2_. The inoculum was removed, cells washed twice, and infection left to progress in serum-free DMEM.

### Endoglycosidase H and PNGase F treatment

Cell lysates, ultracentrifuged medium, and ultracentrifuge pellets were digested with endoglycosidase H (Endo H) or PNGase F (Promega) according to the manufacturer’s instruction. Both digestions were performed for 3 h at 37°C. Digested proteins were subsequently resolved by SDS-PAGE and analyzed by Western blotting.

### LDH release assay

The cytotoxic effect of viral infection on cells was assessed using an LDH release assay, performed according to the manufacturer’s protocol (Roche, Switzerland). To prepare samples for the assay, supernatants were collected and centrifuged at 500 × *g* for 10 min at 4°C to clear cellular debris. The assay was performed in technical replicates in a clear bottom 96-well plate and the absorbance read using a SpectraMax iD3 plate reader (Molecular Devices) at 490 nm with a reference wavelength of 600 nm. Data are presented as a percentage of total possible LDH release.

### Western blotting

Cell lysates were prepared by lysing cells in RIPA lysis buffer (50 mM Tris [pH 7.4], 150 mM NaCl, 0.5% sodium dodecyl sulfate, 0.5% sodium deoxycholate, 1% Triton X-100, and 1× -protease inhibitor cocktail [Roche]). Cell medium samples were first centrifuged at 500 × *g* for 10 min at 4°C to remove large cellular debris. Thereafter, the medium was ultracentrifuged for 2 h at 100,000 × *g* at 4°C in a Beckman TLS-55 rotor. Samples for electrophoresis were prepared under reducing conditions and resolved on SDS-PAGE, then transferred onto a nitrocellulose membrane (Bio-Rad). The primary antisera used for immunoblotting were (dilutions in parenthesis); L57 (1:4,000), a rabbit polyclonal antiserum raised against SA11 VP7 isolated from SDS-PAGE (14); polyclonal guinea pig sera raised against NSP5 (1:500) was a generous gift from Oscar Burrone; polyclonal rabbit antisera against NSP4 (1:4,000), VP6 (1:2,000) and VP2 (1:2,000) was prepared in-house from antigens purified either from recombinant *E. coli* (17), recombinant Sf9 cells (anti-VP2) or VP6 derived from purified DLPs (anti-VP6). G42 (1:2000) is a rabbit polyclonal antiserum prepared in-house against SA11-infected MA104 cells. After washing, membranes were incubated with HRP-conjugated anti-mouse or anti-rabbit IgG antibodies (1:2,000–1:5,000; Sigma Aldrich). Protein bands were visualized using Clarity Western ECL Substrate (Bio-Rad), and images were acquired using an Amersham Imager 600 (GE Healthcare).

### Immobilized lectin binding

Agarose-immobilized lectins including concanavalin A (Con A; GE Healthcare) and wheat germ agglutinin lectin from *Triticum vulgaris* (WGA; Sigma Aldrich) were washed in binding buffer (20 mM Tris-HCl pH 7.4, 0.5 M NaCl, 1 mM CaCl_2_) and added as a slurry to a microfuge tube before addition of ultracentrifuged supernatant and incubated for 60 min at room temperature. The unbound fraction was removed, beads washed with binding buffer, then resuspended in 2× Lamelli Buffer and boiled for 5 min at 95°C. Binding of sVP7 to lectins was determined via western blotting.

### Growth of Caco-2 cells on Transwell filters

Caco-2 cells were seeded at 1 × 10^5^ cells/cm^2^ on 0.4-µm cell culture inserts (Falcon). The cell monolayer was monitored by transepithelial electrical resistance (TEER) measurements taken using chopstick electrodes with a EVOM2 epithelial voltmeter (World Precision Instruments). Media were replaced every 48 h. Cells were infected apically with the SA11 strain of rotavirus at an MOI of 5. Culture medium from the apical and basolateral compartments was harvested at 16 h post infection (hpi).

Distribution of VP6, VP7, and NSP4 was assessed using Western blotting. The titer of infectious viral particles in each compartment was assessed using FFA. Permeability of cell layers at 16 hpi was determined using fluorescein isothiocyanate (FITC) labeled dextran (4 or 250 kDa). Tight junction formation was assessed at 16 hpi by immunofluorescence (IF) with anti-ZO-1 (1:500; BD Biosciences) followed by Alexa Fluor 488-conjugated anti-mouse (1:1,000; Invitrogen) antibodies. Images were taken on a Nikon TiE inverted fluorescent microscope using Andor Clara camera (1.4 MP CCD) and Nikon Elements software.

### Immunoprecipitation of sVP7 with immobilized monoclonal antibodies

mABs 159 and 4C3 were a generous gift from Harry Greenberg, Stanford University. Ascites fluid containing either mAB 159 or mAb4C3 was diluted 1:1 in binding buffer (50 mM sodium borate pH 8.0, 150 mM NaCl, 1 mM CaCl_2_) and incubated with pre-washed protein A/G Sepharose beads on an end-over-end rotating mixer (Labnet Revolver) for 1 h at room temperature. Following incubation, the beads were collected by centrifugation at 300 × *g* for 1 min and the unbound fraction removed. The beads were washed using wash buffer (50 mM sodium borate pH 8.0, 500 mM NaCl, 1 mM CaCl_2_) until A_280_ nm reached a baseline. Medium containing sVP7 was diluted 1:1 in binding buffer and incubated with mAB bound protein A/G beads for 1 h with end-over-end mixing at room temperature. Following incubation, the unbound fraction was removed, and the beads washed as before. For analysis of bound sVP7, beads were boiled in Laemmli buffer for 5 min at 95°C and protein expression analyzed using SDS-PAGE and Western blotting.

### Purification of secreted VP7 and NSP4

Caco-2 cells were seeded into 15 cm^2^ plastic cell culture dishes, grown to confluence, and maintained for a further 14 days to enable differentiation. Cells were infected with trypsin-activated rotavirus strain SA11 at an MOI of 5 for 1 h, after which the inoculum was removed and replaced with fresh DMEM. At 40 hpi, the medium was collected and centrifuged at 100,000 x *g* for 2 h to remove virions and cellular debris. The clarified supernatant was then concentrated using a stirred ultrafiltration cell (Amicon) with a 10 kDa MWCO membrane. Sequential lectin affinity chromatography was employed to separate and purify secreted VP7 and NSP4. The concentrated medium was first applied to a ConA affinity column. NSP4 bound to the ConA matrix, while VP7 remained in the flowthrough, thereby separating the two viral proteins. NSP4 purification was performed as previously described (18), briefly NSP4 was eluted from the ConA affinity column, then further purified by cation exchange (Mono S) chromatography. For VP7 purification, the flowthrough from the ConA column was applied to a WGA affinity column. Bound VP7 was eluted and subsequently purified by anion exchange (Mono Q) chromatography followed by size exclusion chromatography (SEC) using a Superdex 200 10/30 GL column. The purity of VP7 was confirmed by SDS-PAGE with Coomassie Brilliant Blue staining.

### N- and O-glycan release from VP7 protein

Glycan profiling of the isolated VP7 protein was performed in three technical replicates using a well-established glycomics protocol (19–21). In brief, an activated 0.45 μm PVDF membrane (Merck-Millipore) was first cut and placed into the wells of a flat-bottom polypropylene 96-well plate (Corning Life Sciences, Melbourne, Australia). For each replicate, 20 µg of reduced and alkylated VP7 protein was spotted onto the membrane within each well. The membranes were then dried, blocked with 1% (w/v) polyvinylpyrrolidone in 50% (v/v) aqueous methanol, and washed with MilliQ water (used throughout). The *N*-glycans were exhaustively released using 0.5 U/μL recombinant *Elizabethkingia miricola* peptide:*N*-glycosidase F (PNGase F) expressed in *Escherichia coli* (Promega) per well and incubated for 16 h at 37°C. The released *N*-glycans were transferred into fresh tubes and hydroxylated by the addition of 100 mM aqueous ammonium acetate, pH 5 for 1 h at 20°C. The glycans were reduced using 1 M sodium borohydride in 50 mM aqueous potassium hydroxide for 3 h at 50°C. The reduction reaction was quenched using glacial acetic acid. The *O*-glycans were subsequently released from the same protein spots in the 96-well plate by incubation with 20 µL 0.5 M sodium borohydride in 50 mM aqueous potassium hydroxide for 16 h at 50°C. The reaction was quenched using 2 µL glacial acetic acid, and the released and reduced O-glycans were transferred into fresh 1.5-mL Eppendorf tubes.

### Desalting of reduced *N*- and *O*-glycans from VP7 protein

Desalting of the reduced *N*-glycans was performed using porous graphitized carbon (PGC) resin custom packed as micro-columns on top of C18 discs (Merck-Millipore) in P10 solid-phase extraction (SPE) formats (19). For reduced *O*-glycans, dual desalting was performed using firstly strong cation exchange resin (AG 50W-X8 Resin, Bio-Rad) (where the *O*-glycans were not retained), followed by PGC resin (where *O*-glycans were retained) custom packed as microcolumns on top of C18 discs in P10 SPE formats. Following micro-column equilibration and sample loading and washing, the *N*- and *O*-glycans were eluted from the PGC-SPE micro-columns using 0.1% trifluoroacetic acid/50% acetonitrile (ACN)/49.9% water (v/v/v), dried, and resuspended in 20 µL water. Samples were spun at 14,000 × *g* for 10 min at 4°C, and the clear supernatant fractions were carefully transferred to high recovery glass vials (Thermo Fisher Scientific) to avoid debris and particulates in the LC-MS/MS injection vials.

### N- and O-glycan profiling with PGC-LC-MS/MS

The *N*- and *O*-glycans were profiled using a well-established PGC-LC-MS/MS method (19–22). In brief, the glycan samples were injected on a HyperCarb KAPPA PGC-LC column (particle/pore size, 3 µm/250 Å; column length, 30 mm; inner diameter, 1 mm, Thermo Hypersil, Runcorn, UK) heated to 50°C. The glycans were separated over a 60 min linear gradient of 0–45% (v/v) pure ACN (solvent B) in 10 mM aqueous ammonium bicarbonate (solvent A) on a 1260 Infinity Capillary HPLC system (Agilent) operating with a constant flow rate of 20 µL/min. The separated glycans were introduced directly into the mass spectrometer, ionized using electrospray ionization, and detected in negative ion polarity mode using a linear trap quadrupole Velos Pro ion trap mass spectrometer (Thermo Fisher Scientific). The acquisition settings included a full MS1 scan acquisition range of *m/z* 350–2,000, resolution of *m/z* 0.25 full width half maximum (FWHM), and a source voltage of +3.2 kV. The automatic gain control (AGC) for the MS1 scans was set to 5 × 10^4^ with a maximum accumulation time of 50 ms. For the MS/MS events, the resolution was set to *m/z* 0.25 FWHM, the AGC was 2 × 10^4^, and the maximum accumulation time was 300 ms. Data-dependent acquisition was enabled for all samples. The three most abundant precursors in each MS1 full scan were selected for fragmentation using resonance activation (ion trap) collision-induced dissociation (CID) at a normalized collision energy of 33%. Dynamic exclusion of precursors was inactivated. All MS and MS/MS data were acquired in profile mode. The mass accuracy of the precursor and product ions was typically better than 0.2 Da. The LC-MS/MS instrument was tuned and calibrated, and its performance benchmarked using well-characterized bovine fetuin glycan standards analyzed at the same time as the samples of interest.

The generated LC-MS/MS raw data files (made publicly available via GlycoPOST [23], accession number GPST000582) were browsed, interrogated, and manually annotated using Xcalibur v2.2 (Thermo Fisher Scientific), GlycoMod and GlycoWorkBench v2.1 as previously described (22, 24). Briefly, glycans were identified based on the monoisotopic precursor mass, the match between the observed and theoretical MS/MS fragmentation pattern *in silico* generated using GlycoWorkBench, and the relative and absolute PGC-LC retention time of each glycan. The relative abundances of the confidently identified *N*- and *O*-glycans were determined from area-under-the-curve (AUC) measurements based on extracted ion chromatograms performed for all relevant charge states of the monoisotopic precursor *m/z* using RawMeat v2.1 (Vast Scientific) and Skyline (64-bit) v24.1 (25, 26).

### HEK-Blue SEAP assay

Suspensions of HEK-Blue -null, -TLR2, and -TLR4 were adjusted to 2.8 × 10^5^ cells/mL, and 180 µL of each cell suspension added to a 96-well cell culture plate containing mock treated, sVP7, sNSP4, or positive control ligands PAM2 or LPS. Cells and stimulant were left to incubate for 12 h at 37°C + 5% CO_2_. After incubation, the absorbance was read at 630 nm (SpectraMax iD3; Molecular Devices).

### Whole blood activation assay

Consented healthy adult donors (*n* = 4) were bled in accordance with Helsinki declaration ethics under ethics application 028315 via the University of Auckland human participants ethics committee (UAHPEC). Fresh whole blood was aliquoted into round bottom polypropylene tubes. Stimulation of whole blood with purified sNSP4, purified sVP7, buffer control, and PAM2 was performed in a matched volume in triplicate. For all samples, an unstained control was included to account for sample autofluorescence. Tubes were incubated at 37°C for 6 h. Following incubation, cells were blocked with True-Stain FcX Blocker (Biolegend) and True-Stain Monocyte Blocker (Biolegend) for 10 min at room temperature. The full-stain antibody cocktail (Table S1) was prepared in Brilliant Buffer Plus (BD Bioscience), and cells were incubated with antibodies for 20 min at room temperature, protected from light. Red blood cells were then lysed using the BD FACS lyse solution for 5 min. Following red blood cell lysis, all samples were centrifuged at 500 x *g* for 5 min and subsequently washed twice with FACS buffer (2 mM EDTA, 1% human serum, PBS). Samples were acquired on a five-laser Cytek Aurora following spectral unmixing of single-color controls. Data analysis was performed using cloud-based OMIQ software (Dotmatics) (See Fig. S1 and S2 for representative gating strategy and expression of activation markers).

## RESULTS

### Secretion of VP7 from RV-infected cells

**Fig 1:**
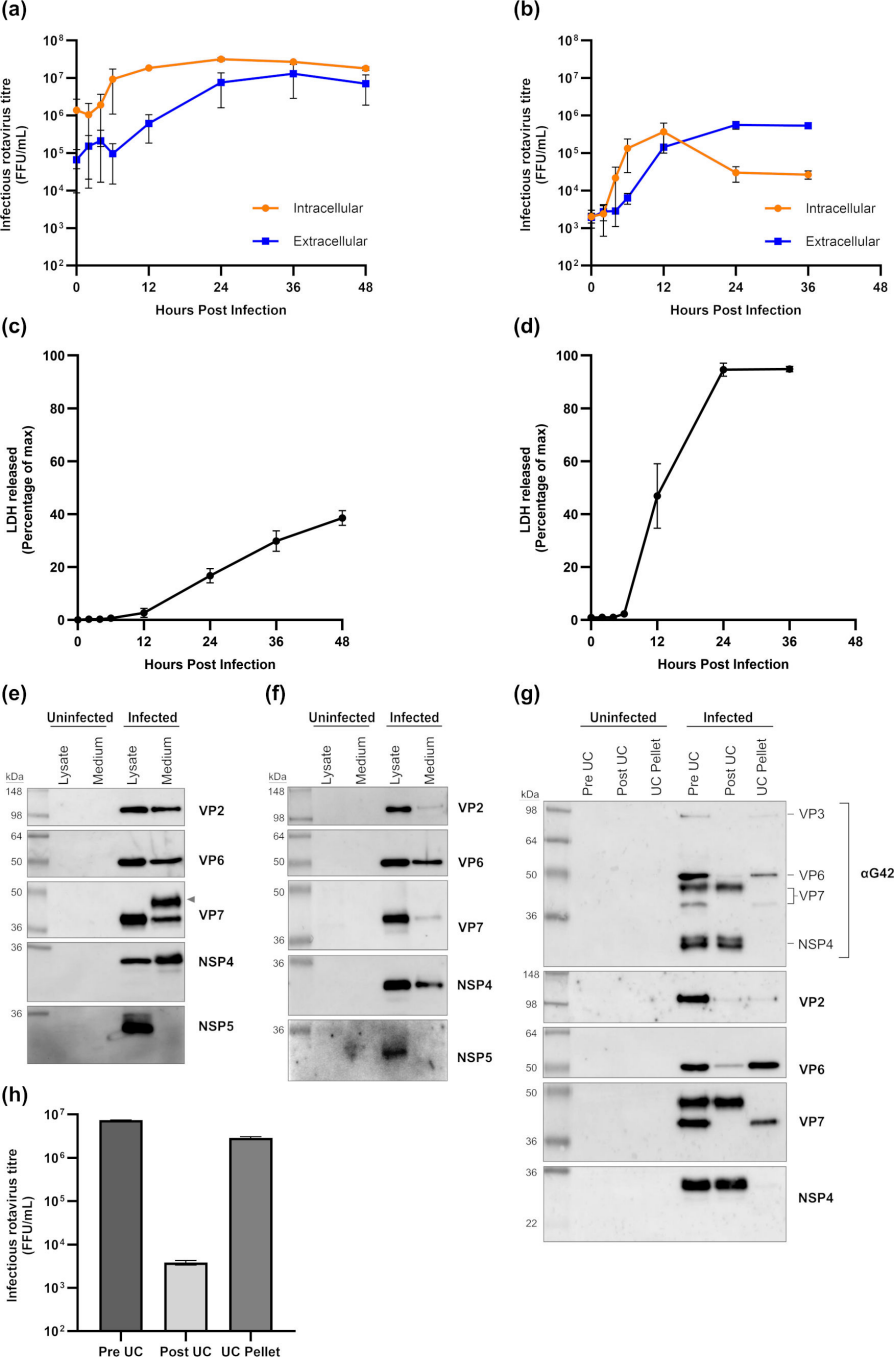
A high molecular weight form of VP7 is secreted from RV-infected Caco-2 cells independently of virions. Caco-2 or MA104 cells were infected with SA11 rotavirus (RV) at an MOI of 5. Viral replication kinetics in Caco-2 (**a**) and MA104 (**b**) cells were determined by titration of intracellular and extracellular virus using a focus forming assay (FFA). Cell viability in Caco-2 (**c**) cells and MA104 (**d**) cells was measured by assaying the amount of LDH in the cell media. The distribution of RV proteins in the medium and lysate of RV-infected Caco-2 cells at 24 hpi (**e**) and MA104 cells at 12 hpi (**f**) was resolved by immunoblotting. Arrow indicates the high molecular weight form of VP7. To determine if the secretion of VP7 occurred independently from the virion, the distribution of virions and high MW VP7 was compared before and after ultracentrifuge of cell medium. Cell medium (Pre UC), ultracentrifuged medium (Post UC), and ultracentrifuged medium pellet (UC pellet) fractions were titrated by FFA (**g**). Distribution of viral proteins across these fractions was analyzed by immunoblotting (**h**).

Previous studies have demonstrated that the viability, as measured by plasma membrane permeability, of polarized Caco-2 cells is maintained for at least 24 h following synchronous infection with rotavirus, while infected MA104 cells undergo complete lysis within this period (16, 27). We sought to determine whether the prolonged viability of infected Caco-2 cells led to different fates of rotavirus proteins compared with those observed in MA104 cells. Both cell types were infected with the SA11 rotavirus (MOI of 5), and infection was left to proceed for up to 48 h. Within this period, the amount of intracellular and extracellular virus was measured, and plasma membrane integrity was assessed by monitoring the release of cytoplasmic lactate dehydrogenase (LDH) (Fig. 1a through d). The distribution of rotavirus proteins in the cell lysate and medium fractions was also analyzed by immunoblotting. We examined the distribution of three structural proteins, VP2, VP6, and VP7, each representative of a different layer in the triple-layered virion. Additionally, we examined the distribution of two non-structural proteins: NSP5, a component of viroplasms, and the non-structural glycoprotein NSP4. The three structural RV proteins, and NSP4, could be detected in both the lysate and medium fractions in both types of infected cells, although relative amounts differed between proteins and cell type (Fig. 1e and f). Notably, a high molecular weight form of VP7 was detected in the medium of infected Caco-2 cells but not in infected MA104 cells (arrowhead, Fig. 1e).

Secretion of infectious virus from infected Caco-2 cells approached maximal levels at 24 hpi (Fig. 1a) when the integrity of the plasma membrane was largely intact (Fig. 1c); therefore, the presence of RV structural proteins in the medium could represent release of virions or alternatively the active secretion of proteins from infected cells, independently of virions. To distinguish between these two possibilities, the distribution of virions and viral proteins was compared before and after ultracentrifugation of medium collected from RV-infected Caco-2 cells at 24 hpi (Fig. 1g and h). As expected, virion distribution following ultracentrifugation revealed that >99.9% of infectious virus was depleted from the medium. A similar distribution was observed for the major structural protein VP6 and the lower MW form of VP7. In contrast, both the higher MW form of VP7 and NSP4 were recovered exclusively in the ultracentrifugation supernatant, indicating they were secreted from infected cells independently of virions.

### Modification of N-linked glycan on secreted VP7

**Fig 2:**
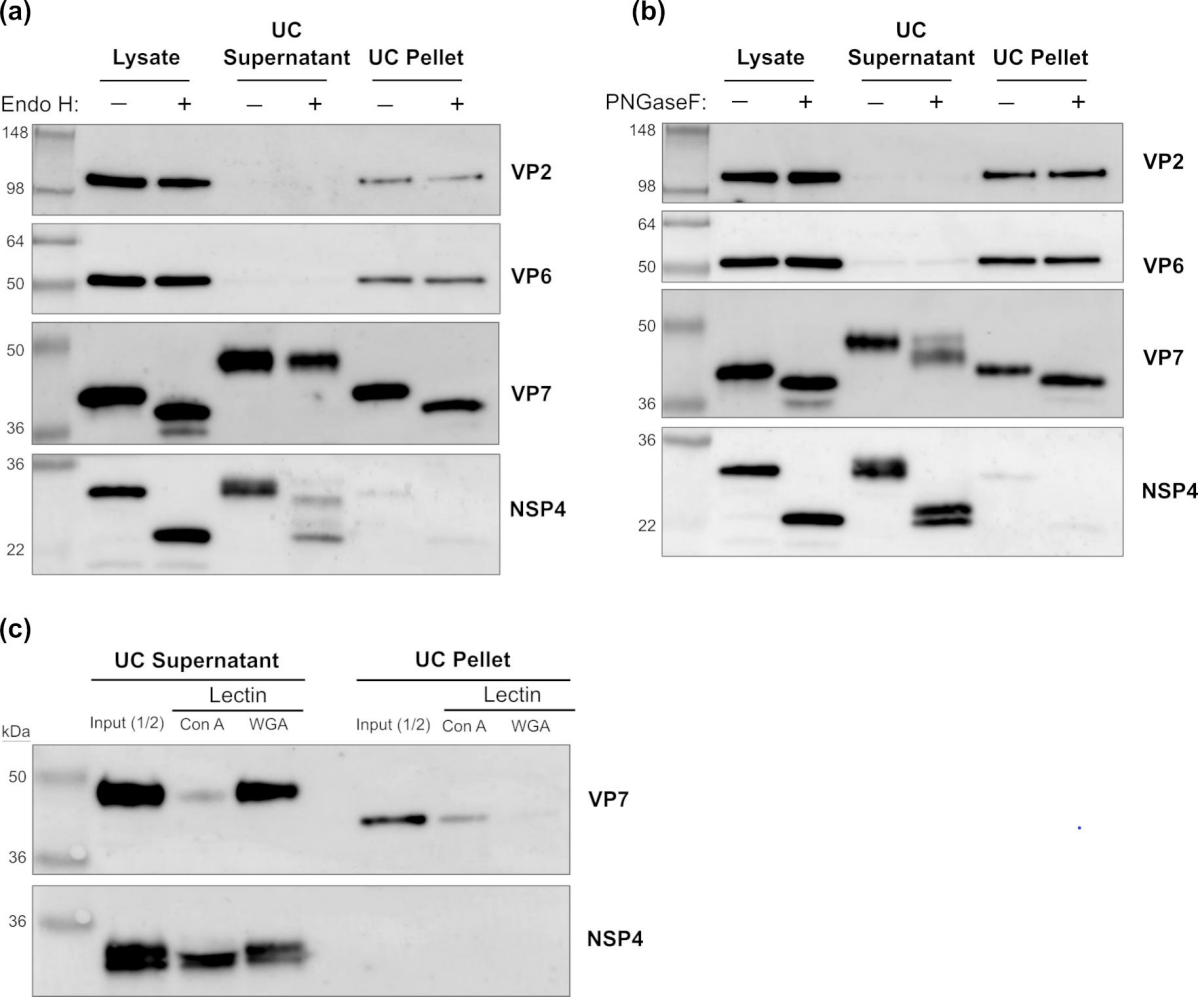
Enzymatic deglycosylation reveals a different pattern of N-linked glycosylation in sVP7 compared with cell and virion-associated VP7. Caco-2 cells were infected with SA11 RV (MOI = 5) for 24 h, after which the medium was removed and ultracentrifuged (UC) producing UC supernatant and UC pellet fractions. These fractions were incubated with EndoH (**a**) and PNGaseF (**b**) as described in Materials and Methods and viral protein expression detected by SDS-PAGE and immunoblotting. The binding of secreted VP7 and NSP4 to immobilized ConA and WGA was performed. Binding of VP7 or NSP4 from the UC supernatant and UC pellet to either lectin was analyzed by immunoblotting (**c**).

The high MW secreted form of VP7 (hereafter referred to as sVP7) observed in our experiments has not been reported previously in any study of rotavirus infection to our knowledge. We considered that the increased molecular weight was potentially due to modification of the N-linked glycan attached to Asn69 in the ER and/or potentially to additional post-translation modifications not previously identified. Samples corresponding to the infected cell lysate and both the pellet and supernatant fraction of ultracentrifuged infected cell medium were subjected to enzymatic digestion with endoglycosidase H (EndoH) and PNGase F. Subsequent immunoblot analysis was employed to assess the status of N-linked glycans attached to rotavirus proteins. Both EndoH and PNGase F reduced the MW of VP7 and NSP4 present in cell lysates (Fig. 2a and b), indicating that these glycoproteins contained only oligomannosidic-type N-linked glycans typical of glycoproteins that are retained in the ER. EndoH also reduced the MW of VP7 associated with the virus particles that were recovered in the ultracentrifuge pellet (Fig. 2a), indicating that the single N-linked glycan present on VP7 remains unmodified during the assembly of VP7 into the outer capsid layer and subsequent release of virus from cells. In contrast, no reduction in MW was observed in sVP7 present in the supernatant after ultracentrifugation of medium following EndoH treatment (Fig. 2a). A reduction in MW was observed in sVP7 following treatment with PNGase F, but this enzyme did not reduce the apparent MW of the protein to the same size as the PNGase F-treated VP7 present in cell lysate or ultracentrifuge pellet fractions (compare position of bands indicated by open and closed arrowheads, Fig. 2b). As observed previously, the secreted form of NSP4, which contains two N-linked glycans, exhibited only partial sensitivity to endoH, while PNGase F treatment did not reduce the MW to the size of the unglycosylated form of NSP4 evident after endoglycosidase digestion of the cell-associated form of this glycoprotein (16). These results suggest that the single N-linked glycan attached to sVP7 is modified, most likely during secretion through the Golgi complex, rendering it insensitive to EndoH.

Glycosylation was also examined by comparing the lectin-binding profile of each secreted rotavirus glycoprotein. Concanavalin A (ConA), a lectin with affinity for mannose-rich glycans and wheat germ agglutinin (WGA), which preferentially binds to N-acetyl glucosamine, linked to agarose beads, was used to capture the secreted glycoproteins present after ultracentrifugation of infected cell medium (Fig. 2c). sVP7 bound strongly to WGA but only weakly to ConA while secreted NSP4 bound to both immobilized lectins with similar efficiency. Though the quantities available for this experiment were limiting, the VP7 associated with the virion exhibited an apparent preference for ConA binding in contrast to the secreted form. These results are consistent with endoglycosidase sensitivity of the different glycoproteins and further suggest that the glycan attached to sVP7 is modified from a high-mannose form during secretion.

### Secretion of sVP7 is inhibited by drugs that impair Golgi function

**Fig 3:**
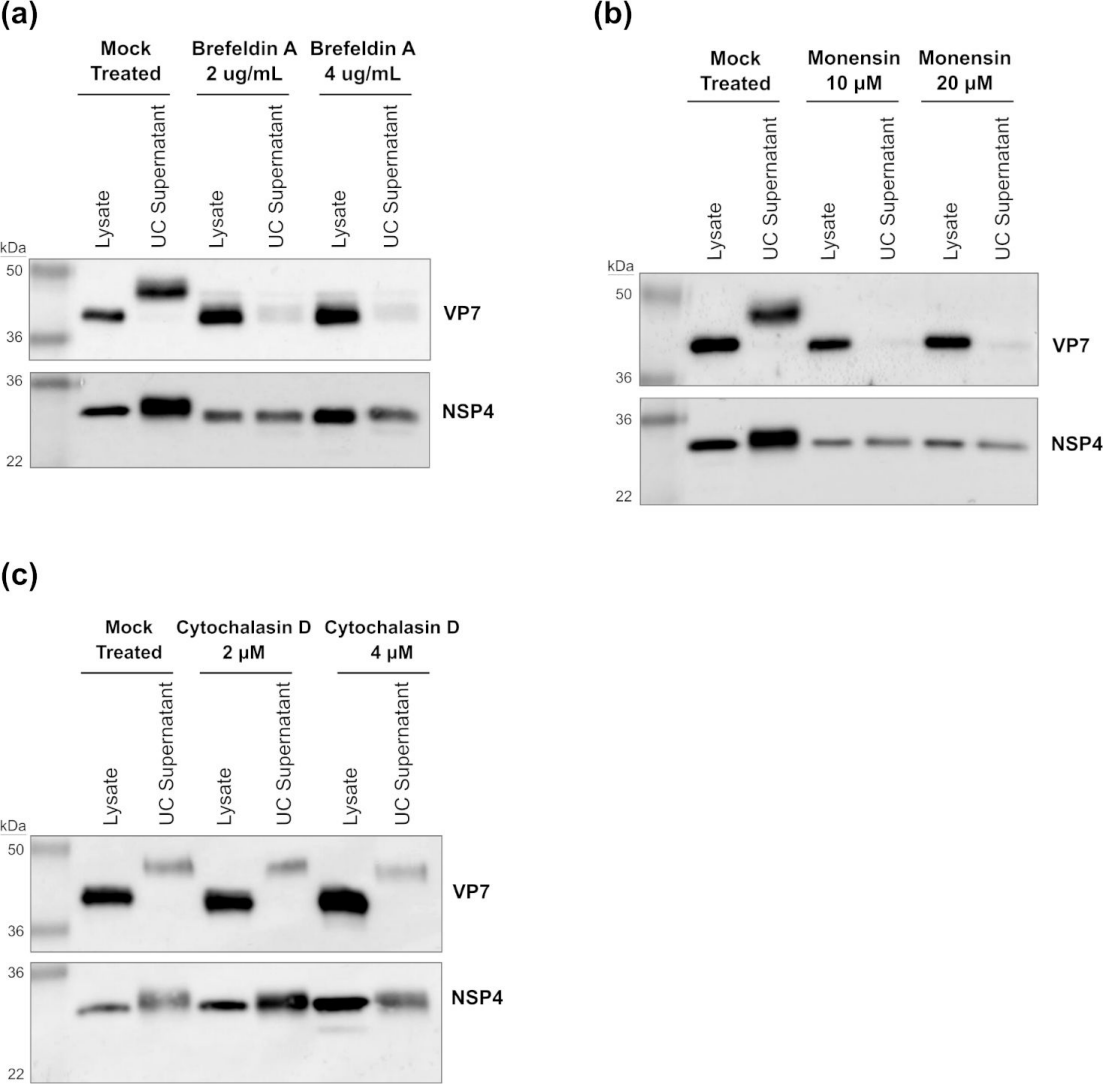
Secretion of VP7 is inhibited by drugs that impair Golgi function. Caco-2 cells were infected with SA11 RV (MOI = 5). Infected cells were treated with BFA (**a**), Monensin (**b**), and Cytochalasin D (**c**) at the indicated concentrations to the medium at 1 hpi. At 16 hpi, cell lysates and UC supernatant were prepared to analyze the effect of the compounds on secretion of RV proteins by SDS-PAGE and immunoblotting.

Modification of the N-linked glycan present on sVP7 suggested this form of the protein could be transported into the medium via the conventional secretory pathway involving transit through the Golgi complex. To investigate this possibility, we examined the effect of drugs that impair Golgi function and inhibit the secretory process. Brefeldin A (BFA) redistributes *cis* Golgi elements into the ER, whereas monensin blocks trafficking of vesicles from the *trans* Golgi network (28, 29). Both drugs prevented the detection of sVP7 in the ultracentrifuged medium of infected cells at a concentration that was neither cytotoxic (data not shown) nor reduced the level of VP7 expressed within infected cells (Fig. 3a and b) (16). Secretion of sVP7 was not impaired when infected cells were treated with cytochalasin D, which disrupts the structure of actin microfilaments (Fig. 3c) (30).

### Polarized secretion of sVP7 from rotavirus-infected cells

**Fig 4:**
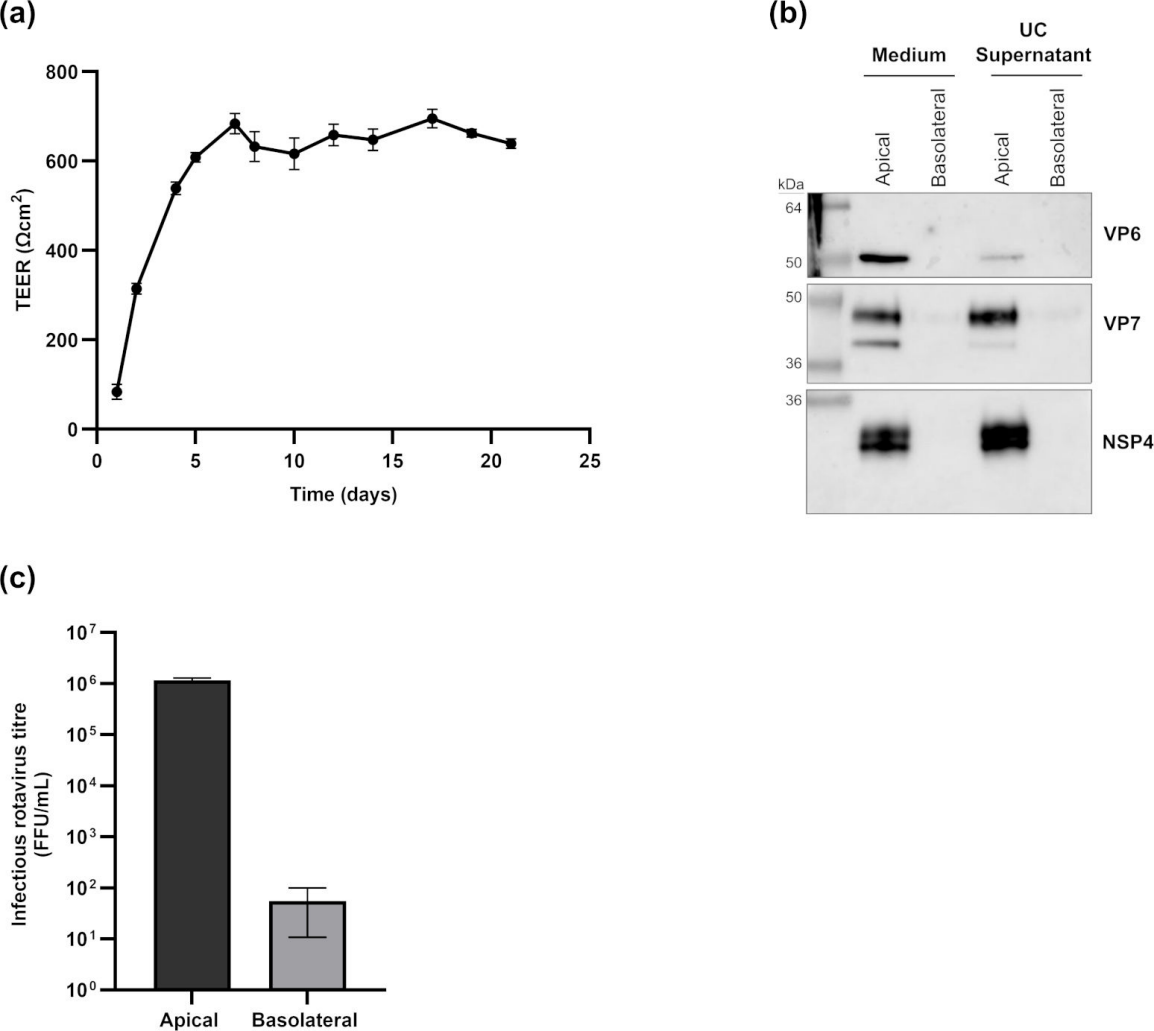
VP7 is secreted from the apical surface of polarized Caco-2 cells. Caco-2 cells were seeded on 0.4-µm pore Transwell inserts and cultured for 21 days, during which TEER was monitored to confirm formation of an intact epithelial barrier (**a**). Cells were apically infected with SA11 RV (MOI = 5), and medium was collected from both the apical and basolateral compartments. RV protein present in the media was analyzed by SDS-PAGE and immunoblotting both before and after ultracentrifugation of the medium (**b**). The distribution of infectious RV released from the cells was examined by titration of the medium prior to ultracentrifugation (**c**). All experiments were done as three biological repeats.

Previous studies have reported that RV infection of uniformly polarized Caco-2 cells grown on semipermeable filters results in the selective release of virions from the apical surface (11, 16). The secretion of NSP4 also exhibits a similar polarity (16). To investigate whether sVP7 followed the apical secretion pathway of virions and NSP4, cells were seeded onto semipermeable filters and grown for 21 days, at which time the transepithelial electrical resistance (TEER) reached a plateau indicating formation and maintenance of a uniformly polarized monolayer (Fig. 4a). Cells were infected by addition of RV to the apical surface, and the apical and basolateral medium collected and analyzed for the presence of infectious virus, NSP4, and VP7 (Fig. 4b). The results confirm that sVP7 undergoes polarized secretion into the apical medium. Junctional integrity at the time of sample collection was confirmed by paracellular diffusion of 4 and 250 kDa fluorescent dextran as well as imaging of tight junctional protein, ZO-1 (Fig. S3).

### Recognition of sVP7 by conformation-dependent neutralizing antibodies

**Fig 5:**
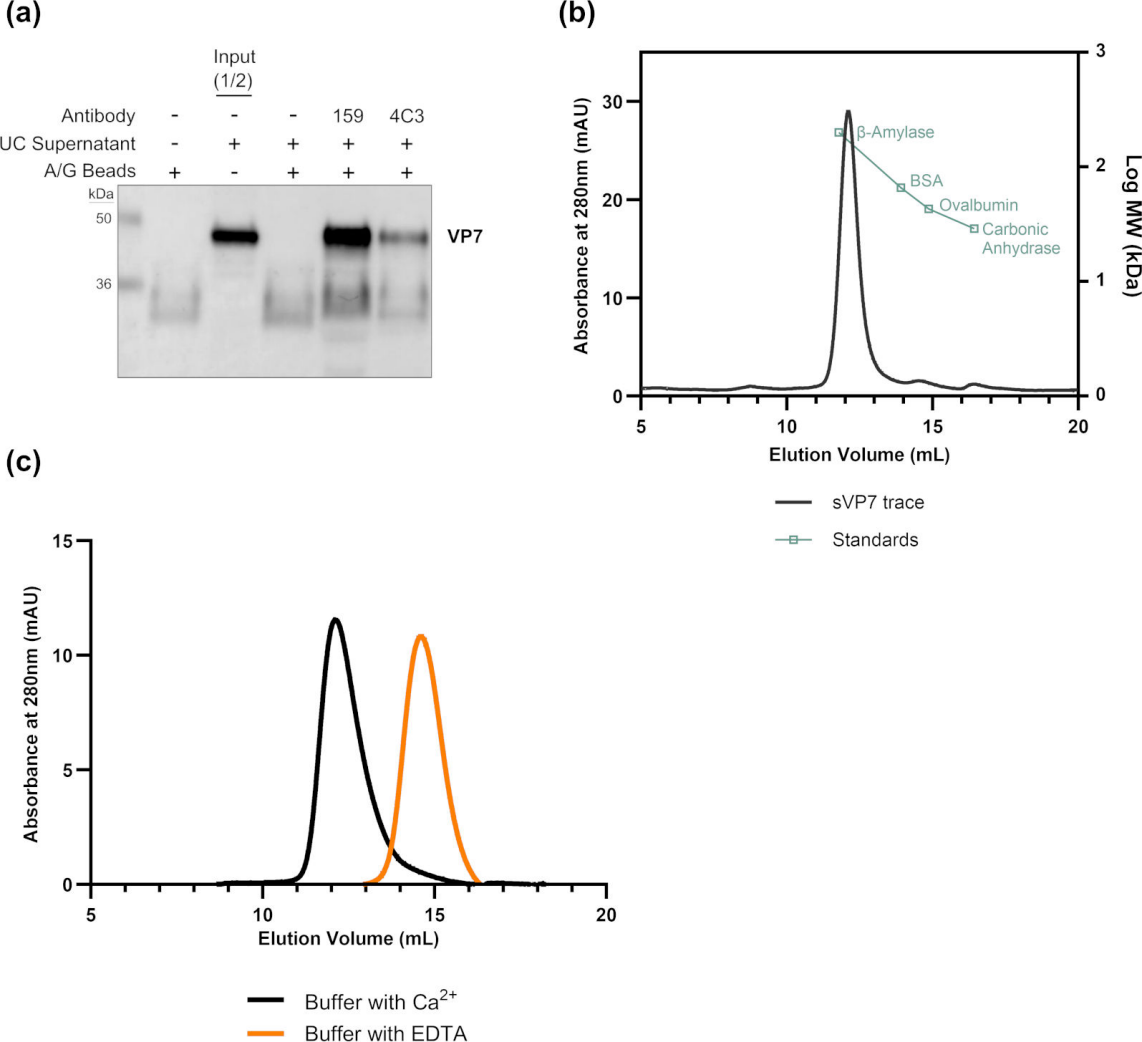
sVP7 is recognized by conformation-dependent neutralizing monoclonal antibodies and is secreted as a calcium-dependent trimer. High MW VP7 bound by rotavirus neutralizing monoclonal antibodies (mAB) targeting trimeric VP7 found on the surface of the virion. mAB 159 and 4C3 were immobilized onto protein A/G beads before being incubated with ultracentrifuged cell medium from rotavirus infected Caco-2 cells. Bound proteins were boiled from the mAB protein A/G bead complex and the presence of high MW VP7 determined by SDS-PAGE and immunoblotting (**a**). SEC of high MW VP7 was performed using a Superdex 200 10/300 GL (**b and c**). A Superdex 200 10/300 GL column was calibrated with β-amylase (200 kDa), BSA (66 kDa), ovalbumin (43 kDa), and carbonic anhydrase (29 kDa). The size of high MW VP7 (sVP7) purified by a combination of lectin affinity chromatography and ion exchange chromatography was investigated by SEC under the same conditions as the protein standards to allow for comparison (**b**). Following SEC, the eluted sVP7 protein was reanalyzed by SEC under two different buffer conditions, buffer containing Ca^2+^ or buffer containing EDTA (**c**).

The detection of VP7 in immunoblots relies on the use of a polyclonal VP7 antibody that can detect denatured forms of the protein following SDS-PAGE. To confirm that sVP7 retained conformation-dependent epitopes that exist within the outer capsid layer of the virion, we examined the ability of two neutralizing monoclonal antibodies (mAbs) specific for VP7 to bind to the secreted form of the protein. Each mAb was immobilized on Protein A/G beads and added to a volume of medium collected from RV-infected cells after ultracentrifugation. The beads were collected, washed, and bound proteins were eluted by boiling in 2× Laemmli buffer and analyzed by immunoblot (Fig. 5a). The results show that mAb 159 and 4C3 both recognize sVP7, indicating that the conformational neutralizing epitopes present in the outer capsid of the virion are retained in the secreted form of the protein.

Previous investigation has revealed that the recognition of VP7 by conformationally sensitive mAbs is dependent upon the formation of a trimer (31, 32). To confirm that the sVP7 can form trimers independently of its assembly onto the surface of virions, we examined the size of the secreted protein following its purification from the medium of RV-infected cells by size exclusion chromatography (SEC). Prior to analysis by SEC, sVP7 was purified from ultracentrifuged medium of infected cells and purified via immobilized lectin (WGA) affinity chromatography followed by ion exchange chromatography (Mono Q). Fractions eluted from Mono Q containing sVP7 were pooled and applied to a Superdex 200 10/30 GL column. The elution profile observed indicated that sVP7 was homogeneous with a K_av_ = 0.233, corresponding to an apparent MW of 161.9 kDa (Fig. 5b). This value is higher than the apparent MW of 141 kDa determined for trimeric recombinant VP7 purified by immunoaffinity chromatography (33) but remains consistent with a trimeric structure, albeit with additional mass putatively due to further post-translational modifications.

During rotavirus entry, uncoating of VP7 from the viral particle is triggered by the loss of Ca^2+^ from the uptake vesicle (34). Ca^2+^-dependent trimerization is similarly evident in recombinant VP7 (33). To determine if the sVP7 trimer is also stabilized by Ca^2+^, SEC was performed in the presence of Ca^2+^ or EDTA (Fig. 5c). Following SEC on a Superdex 200 calibrated with Ca^2+^, the purified sVP7 eluted with a K_av_ of 0.233, corresponding to the aforementioned apparent MW of 161.9 kDa. When the SEC was performed on a Superdex 200 calibrated with buffer containing EDTA, the purified sVP7 eluted with a K_av_ of 0.394, corresponding to an apparent molecular weight of 54.4 kDa. These results confirmed sVP7 is secreted as a calcium-dependent trimer.

### Glycan profiling of sVP7

**Fig 6:**
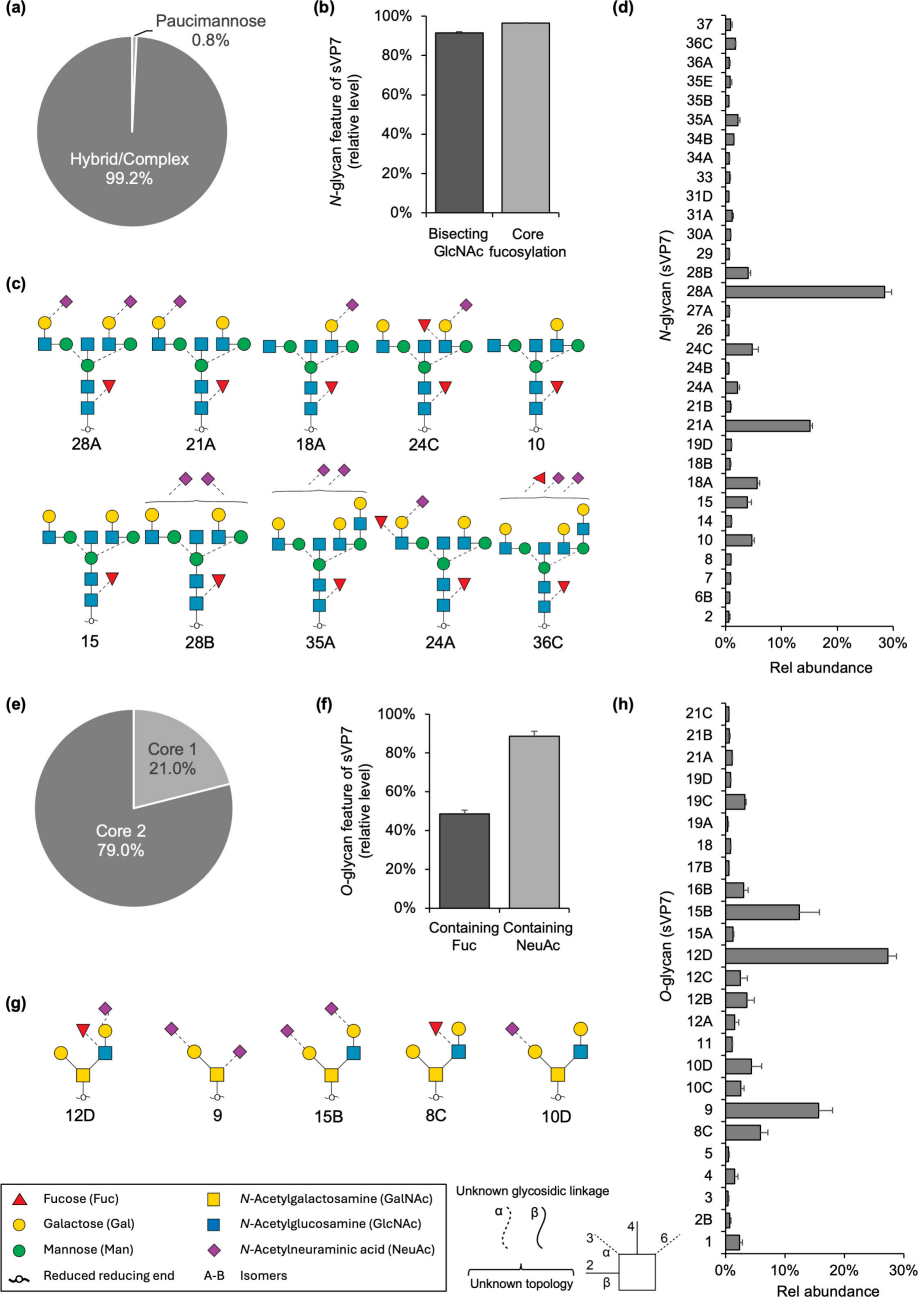
sVP7 glycosylation. Distribution of *N*-glycan types decorating the sVP7 protein (**a**). Levels of bisecting GlcNAc and core fucosylation of the identified sVP7 *N*-glycans (**b**). Ten most abundant sVP7 *N*-glycoforms, depicted using the latest symbol nomenclature for glycans (SNFG) (**c**). Relative abundance (mean ± SD, *n* = 3 technical replicates) of individual *N*-glycan structures identified on sVP7 (**d**). Distribution of *O*-glycan core types decorating the sVP7 protein (**e**). Relative levels of sialylation and fucosylation among the identified sVP7 *O*-glycans (**f**). Five most abundant sVP7 *O*-glycoforms (**g**). Relative abundance (mean ± SD, *n* = 3 technical replicates) of individual *O*-glycan structures identified on sVP7 (**h**). Note, low abundance N- and O-glycans less than 0.5% relative abundance have been omitted in these graphs; see Table S2 for all identified glycan structures.

Determination of the glycan structures associated with sVP7 was performed using a well-established LC-MS/MS-based glycomics protocol (19). Glycoprofiling of the sVP7 identified a total of 70 N-glycan structures almost exclusively of the complex type (Fig. 6a, see Table S1 for all tabulated N-glycan data). The sVP7 *N-*glycans were abundantly modified with bisecting GlcNAc and core fucosylation, known features of differentiated Caco-2 cells (Fig. 6), (35). The dominance of these structural features suggests that FUT8 and MGAT3, the two glycoenzymes responsible for core fucosylation and bisecting GlcNAc, readily act on sVP7 as the virion passes through the medial-Golgi. While a minor proportion of paucimannosidic N-glycans were observed on sVP7, no oligomannosidic-type N-glycans were observed, consistent with the lack of EndoH sensitivity. The most prevalent N-glycans carried by sVP7 were of the bi-antennary type and were capped with terminal α2,6 sialylation, e.g. glycan 28A and 21A (Fig. 6c and d).

Surprisingly, the glycoprofiling also revealed that mucin-type O-glycans are linked to sVP7. A total of 41 O-glycan structures, most prominently core one or two, were identified (Fig. 6e, see Table S2 for all tabulated O-glycan data). Most of the sVP7 O-glycans were capped with sialylation (~85%) and/or fucosylation (~45%) (Fig. 6f). Despite considerable molecular heterogeneity, the five most abundant sVP7 O-glycan structures were found to account for almost two-thirds of the O-glycan molecules linked to the virion (Fig. 6g through h). While site-specific analyses are still required to determine the exact location(s) of the O-glycans on the sVP7 protein structure, the presence of virion protein O-glycosylation is interesting as it suggests functional roles of these previously overlooked carbohydrate chains and may serve to explain the unexpectedly high MW glycoform of sVP7 after PNGase F treatment (Fig. 2b).

### Activation of TLR2 and TLR4 by sVP7

**Fig 7:**
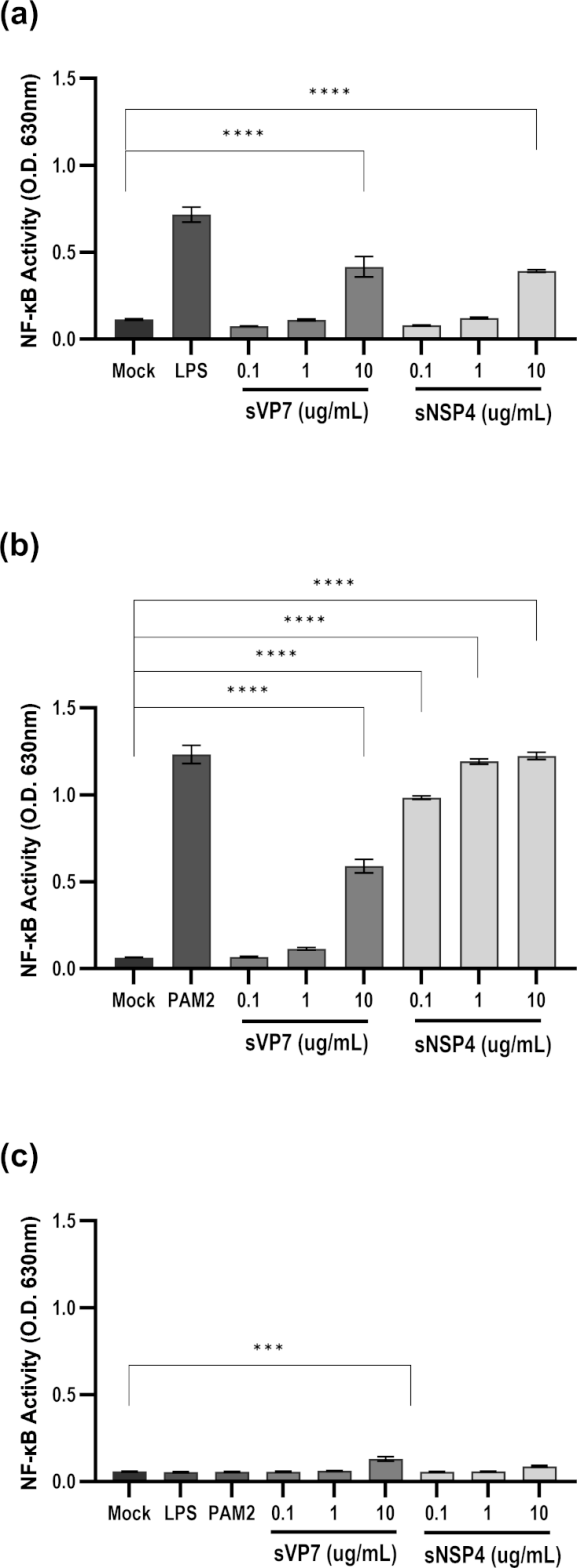
sVP7 is an agonist of TLR2 and TLR4 signaling in HEK-Blue cells. HEK-Blue-TLR2 (**a**), HEK-Blue-TLR4 (**b**), and HEK-Blue-Null (**c**) cells were stimulated with 0.1, 1.0, and 10 µg/mL sVP7 or sNSP4 for 12 h. Pam2CSK4 (PAM2; 10 ng/mL) and lipopolysaccharide (LPS; 10 ng/mL) were used as positive control agonists for TLR2 and TLR4 stimulation, respectively. The activation of NF-κB was monitored by SEAP activity measured at 630 nm. Data represent mean ± SEM of three independent experiments. Statistical significance was determined using two-way ANOVA with Dunnett’s multiple comparison to mock-treated cells (*** *P* < 0.001, **** *P* < 0.0001).

We considered that the novel glycan signature associated with sVP7 may confer biochemical activity consistent with a pathogen-associated molecular pattern (PAMP) consistent with the ability to induce Toll-like receptor (TLR) signaling. This function has previously been described for NSP4, which is a TLR2 agonist (36). To assess the potential for sVP7 to activate the host innate immune response, the ability for the protein to stimulate TLR2 or TLR4 signaling was investigated. Secreted embryonic alkaline phosphatase (SEAP) reporter HEK293T cells expressing TLR2 (HEK-Blue-TLR2), TLR4 (HEK-Blue-TLR4), or neither receptor (HEK-Blue-Null) were mock stimulated or stimulated with sVP7, sNSP4, or the canonical ligands Pam_2_CSK_4_ (PAM2) or lipopolysaccharide (LPS) for TLR2 and TLR4, respectively. This approach confirmed the potent agonism of TLR2 by NSP4 but also revealed activation of NF-κB signaling by sVP7 stimulation in both HEK-Blue -TLR2 and -TLR4 cells, but not HEK-Blue-Null cells at the maximal concentration used in this assay (10 µg/mL) (Fig. 7a through c).

### Innate immune cells within whole blood are activated following stimulation with sVP7

The secretion of a form of VP7 carrying a previously underappreciated glycosylation pattern represents a novel viral ligand capable of interacting with cells not conventionally regarded as permissive for RV infection. The secreted rotaviral glycoprotein, NSP4, was previously identified as a TLR2 agonist capable of inducing secretion of pro-inflammatory cytokines from macrophages (36). Therefore, to assess the immunostimulatory potential of sVP7, we evaluated the activation of key innate immune cell populations within human whole blood using flow cytometry. Whole blood from healthy donors (*n* = 4) was stimulated with sVP7 purified from the medium of RV-infected Caco-2 cells for 6 h, followed by staining for lineage-specific markers and established activation markers. Alongside sVP7, whole blood was also stimulated with purified sNSP4 and the TLR2 agonist Pam_2_CSK_4_ (PAM2).

**Fig 8:**
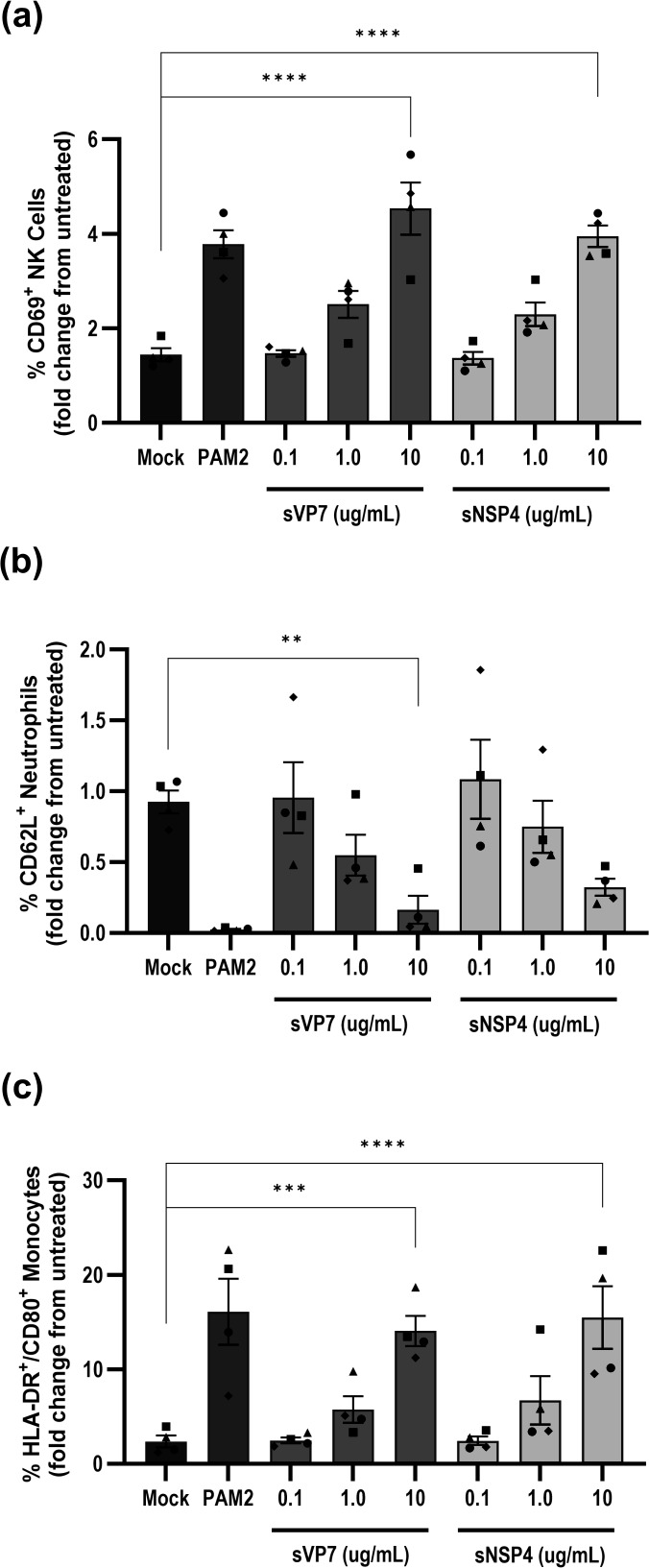
Activation marker expression changes on NK cells, neutrophils, or classical monocytes following stimulation with high MW VP7. Fresh whole blood was stimulated with purified sVP7, purified sNSP4, or PAM2 for 6 h. The response of CD69 on NK cells (**a**), CD62L on neutrophils (**b**), and HLA-DR+/CD80+ on monocytes (**c**) following stimulation is presented as fold change relative to untreated whole blood. Data represent mean ± SEM of three independent experiments. Statistical significance was determined using two-way ANOVA with Dunnett’s multiple comparison to mock-treated cells (*** *P* < 0.001, **** *P* < 0.0001).

Flow cytometry was employed to assess innate immune activation by measuring the expression of activation markers across selected immune cell populations in whole blood. CD80 expression was assessed on classical monocytes (CD14^+^CD16^-^), CD69 on NK cells (CD3^-^CD56^+^), and CD62L on neutrophils (CD15^+^CD16^+^). Following stimulation with purified sVP7, a dose-dependent increase in CD80 expression on classical monocytes and CD69 on NK cells was observed (Fig. 8a and c). Additionally, neutrophil surface expression of CD62L decreased in a dose-dependent manner (Fig. 8b), consistent with CD62L shedding, a known marker of neutrophil activation in response to proinflammatory stimuli. Taken together, these data suggest that sVP7 induces innate immune activation across multiple cell types.

## DISCUSSION

The primary mode by which non-enveloped viruses are released from infected cells was long considered to be due to cell lysis. The availability of cell lines that exhibit characteristics typical of polarized epithelial cells has enabled this view to be revised and has revealed the existence of non-lytic mechanisms of viral egress (12, 37, 38). For rotavirus, these studies have relied mainly on the use of Caco-2, a cell line derived from a human adenocarcinoma that differentiates spontaneously upon reaching confluence forming domes on plastic due to transepithelial fluid movement and the formation of tight junctions (39). Polarized sorting and release of rotavirus *via* the apical surface of infected Caco-2 was observed by Jourdan et al., who proposed a “non-classical” vesicular trafficking pathway that bypassed the Golgi (11). More recent studies have confirmed that rotavirus release from polarized intestinal cells precedes damage to the plasma membrane resulting in release of virus within extracellular vesicles that may serve to shield virions from neutralizing antibodies (12). Collectively, these studies have focused on release of infectious virions, but we have previously demonstrated that NSP4, the nonstructural glycoprotein encoded by RV, also undergoes polarized secretion from infected Caco-2 cells (16). Our study reveals that VP7, the major component of the outer capsid layer of the virion, is also secreted from polarized cells independently of its association with the virion and with hitherto unrecognized post-translational modification.

Expression of VP7 variants truncated at the N-terminus identified a cleavable signal sequence and separate ER retention motif (14, 40). When the cleavable signal sequence of VP7 was replaced with a functionally homologous sequence from influenza hemagglutinin, the resultant protein was secreted from transfected cells, suggesting that interaction between the cleaved signal peptide and other downstream sequences in VP7 was required for retention of the protein in the ER (14). In rotavirus-infected cells, the retention of VP7 in the ER is disrupted during the later stages of virion morphogenesis when the outer capsid layer is assembled onto the surface of the immature double-layered particles (DLPs) in the ER lumen. This process remains one of the least well understood aspects of rotavirus replication and is rare, if not unique, among non-enveloped viruses. Budding of DLPs from cytoplasmic viroplasms into the ER is triggered by their interaction with NSP4 on the cytoplasmic face of the ER membrane. The luminal topology of VP7 necessitates that the envelope acquired by the DLP during budding is transient and is removed to enable contact between VP7 and the underlying VP6 layer on the surface of the DLP (41, 42). This step could potentially be mediated by disruption of the lipid bilayer surrounding the particles by either of the rotavirus glycoproteins, both of which have been shown to possess membrane destabilizing activity (43–45) or by VP4 (42).

The assembly of VP7 to create the outer capsid of the virus involves conformational rearrangement of the protein from its membrane-associated form, which has not been clearly defined, to a well-ordered Ca^2+^-binding trimer (31, 33, 46). The N-terminus of mature VP7 present in the capsid begins at residue Gln51 following removal of residues 1–50, where a cleaved signal peptide is located during translocation into the ER (15). The ~ 30 amino acid sequence at the N-terminus of mature VP7, which includes the single site for N-linked glycans at Asn69, is thought to form a “grip arm” responsible for interactions that stabilize the outer capsid on the virion including the interaction of adjacent trimeric capsomers in the outer layer (31, 46). Significantly, residues located in the grip arm are also implicated in retention of VP7 in the ER, suggesting that a “displacement” of this region could represent a key step in the transition of the protein from a membrane-associated form to the trimeric capsid form.

Our study represents the first report of VP7 secretion as a novel glycoform. sVP7 shares the trimeric structure and conformation-dependent epitopes found within the virion. Therefore, secretion of soluble capsid trimers could represent a strategy for interference in antibody-mediated neutralization of RV in infected hosts. Several viruses release soluble forms of key neutralizing antigens, including the sG protein of respiratory syncytial virus, sGP protein of Ebola virus, and ORF2 protein of hepatitis E virus (47–49). The shared antigenicity between the soluble and virion-associated forms of these proteins allows for soluble viral proteins to act as decoys against neutralizing antibodies produced during infection. The production of neutralizing antibodies against VP7 is well reported during rotavirus infection and primarily functions to prevent virion decapsidation (31, 50). Recognition of the secreted form of VP7 by neutralizing antibodies highlights a potential for this protein to enact this function.

We considered whether sVP7 could exhibit properties unrelated to virus neutralization. Prompted by our previous discovery that secreted NSP4 can act as a pro-inflammatory agonist of TLR2, we compared sVP7 and NSP4 in assays of TLR activation. sVP7 activated TLR2 at the highest concentration (10 µg/mL) only in contrast to NSP4, which was a more potent agonist of this receptor even at 100-fold lower concentration. Interestingly, both sVP7 and NSP4 showed comparable activation of TLR4 at 10 µg/mL. Though clearly less responsive to NSP4 than TLR2 in this assay, the activation of TLR4 by both RV glycoproteins is consistent with other studies that report TLR4 agonism by secreted viral glycoproteins. For example, NS1 secreted by dengue virus activates TLR4 on murine macrophages and human PBMC at concentrations similar to those used in our assay (51). The apparently indiscriminate activation of both TLR4 and TLR2 by sVP7 is also shared by NS1, indicating that viral glycoproteins may exhibit chemical signatures required for activation of multiple receptors (52). However, caution should be exercised in comparing our results with other studies due to the use of the HEK NFκB reporter cell lines expressing TLRs that may be more sensitive to stimulation than primary cells. We also employed a whole blood assay to detect activation of selected innate immune cells, comparing the effects of both sVP7 and NSP4 with a canonical TLR2 ligand. These experiments revealed a stimulation of monocytes, NK cells, and neutrophils through modulation of activation markers caused by both RV glycoproteins. While this experiment does not address the direct response of innate immune cell subsets to sVP7 nor identify specific receptor usage, it is consistent with the identification of proinflammatory signaling identified in NFκB reporter cell lines. Although we did not examine the influence of sVP7 on B cells in this assay, the relatively short window of stimulation would not be appropriate for examining B-cell activation. This is consistent with previous murine models being used to demonstrate VP7 driving polyclonal activation of B cells over a longer stimulation window (53). Additionally, the authors of this study identified that B cell activation was dependent on the conformational integrity of trimeric VP7 but not on VP7 glycosylation and speculated that Toll-like receptor activation by VP7 represented a plausible mechanism for B-cell activation.

Glycoprofiling of sVP7 revealed the presence of complex-type N- and mucin-type O glycans previously unrecognized in rotavirus proteins. The presence of these glycans in sVP7 confers additional prospective immunomodulatory properties through potential interactions with lectin receptors present on innate immune cell subsets. For example, C-type lectin receptors (CLRs) play an important role in shaping antiviral immunity and thus regulating the transmission and dissemination of viruses in the host (54). Further studies are warranted to identify cellular and molecular targets of sVP7 and to dissect the role played by this novel capsid glycoform in shaping rotavirus immunity.

Finally, it should be noted that our results reflect properties of VP7 from SA11, a cell-culture adapted RV strain of simian origin. We acknowledge the potential limitation inherent in using a single cell culture-adapted strain and suggest that further studies are now required to determine whether similar properties are exhibited by VP7 in other RV strains, particularly in human strains. One potentially significant difference between SA11 and many human RV strains is the number of potential N-linked glycosylation sites VP7. While VP7 from SA11 is glycosylated at a single conserved asparagine residue (Asn69), Rotarix, a live attenuated human vaccine strain derived from the G1P[8] virus, encodes a VP7 with two additional N-linked glycosylation sites at position 146 and 238, while VP7 in the attenuated G9P[11] RV strain in Rotovac lacks glycosylation at Asn 69. Whether variability in the glycosylation status of VP7 can influence its secretion and reactivity to innate immune cells should now be investigated.

## Data Availability

The authors declare that the data supporting the findings of this study are available within the article and its supplemental material. The LC/MS glycomics data have been deposited to GlycoPOST (23), under accession number GPST000582.

## SUPPLEMENTAL MATERIAL

Supplemental material jvi.01898-25-s0001.docx Table S1; Fig. S1 to S3.

Table S2 jvi.01898-25-s0002.xlsx Complete list of glycan structures.

ASM does not own the copyrights to Supplemental Material that may be linked to, or accessed through, an article. The authors have granted ASM a non-exclusive, world-wide license to publish the Supplemental Material files. Please contact the corresponding author directly for reuse.
