# Supplementary material for: A novel glycoform of the rotavirus outer capsid protein VP7 is secreted from polarized cells and activates innate immune cells
Source: J Virol. 2026 Feb 24;100(3):e01898-25. doi: 10.1128/jvi.01898-25 (PMC13011347; doi:10.1128/jvi.01898-25)
Supplement: Supplemental material — Table S1; Fig. S1 to S3. [file jvi.01898-25-s0001.docx]

**Supplementary information for: A novel glycoform of the rotavirus outer capsid protein VP7 is secreted from polarized cells and activates innate immune cells.**

Ashley Nutsford^1^, The Huong Chau^2^, Carol Wang^1^, Ash Sargent^1^, Camren Cullen^1^, Thomas Reilly^2^, Morten Thaysen-Andersen^2,3^, Anna E.S. Brooks^1,4^ and John A. Taylor^1^*

^1^ School of Biological Sciences, University of Auckland, Auckland, New Zealand.

^2^ School of Natural Sciences, Macquarie University, NSW-2109, North Ryde, Australia

^3^ Institute for Glyco-core Research, Nagoya University, Nagoya, Aichi, Japan

^4^ Liggins Institute, Grafton Campus, University of Auckland, New Zealand

*Correspondence to: [ja.taylor@auckland.ac.nz](mailto:ja.taylor@auckland.ac.nz)

**Supplementary Table 1.** Flow cytometry panel for assessment of immune cell populations in whole blood (Cytek Aurora 5L).

| **Antibody** | **Fluorophore** | **Clone** | **Supplier** | **Catalogue Number** |
| --- | --- | --- | --- | --- |
| CD14 | BUV737 | M5E2 | BD Horizon | 612763 |
| CD45 | Spark 550 | 2D1 | BioLegend | 368550 |
| CD62L | PerCP-eFlour710 | Dreg56 | eBioscience | 46-0629-42 |
| CD80 | PE-Cy5 | 2D10 | BioLegend | 305210 |
| HLA-DR | PE/Fire810 | L243 | BioLegend | 307683 |
| CD3 | Alexa Fluor 700 | UCHT1 | BioLegend | 300424 |
| CD16 | APC/Fire750 | 3G8 | BioLegend | 302060 |
| CD19 | SparkViolet423 | HIB19 | BioLegend | 302282 |
| CD15 | BV510 | W6D3 | BioLegend | 323028 |
| CD69 | BV711 | FN50 | BioLegend | 310944 |
| CD56 | BV750 | 5.1H11 | BioLegend | 362556 |

**Supplementary Figure 1.**


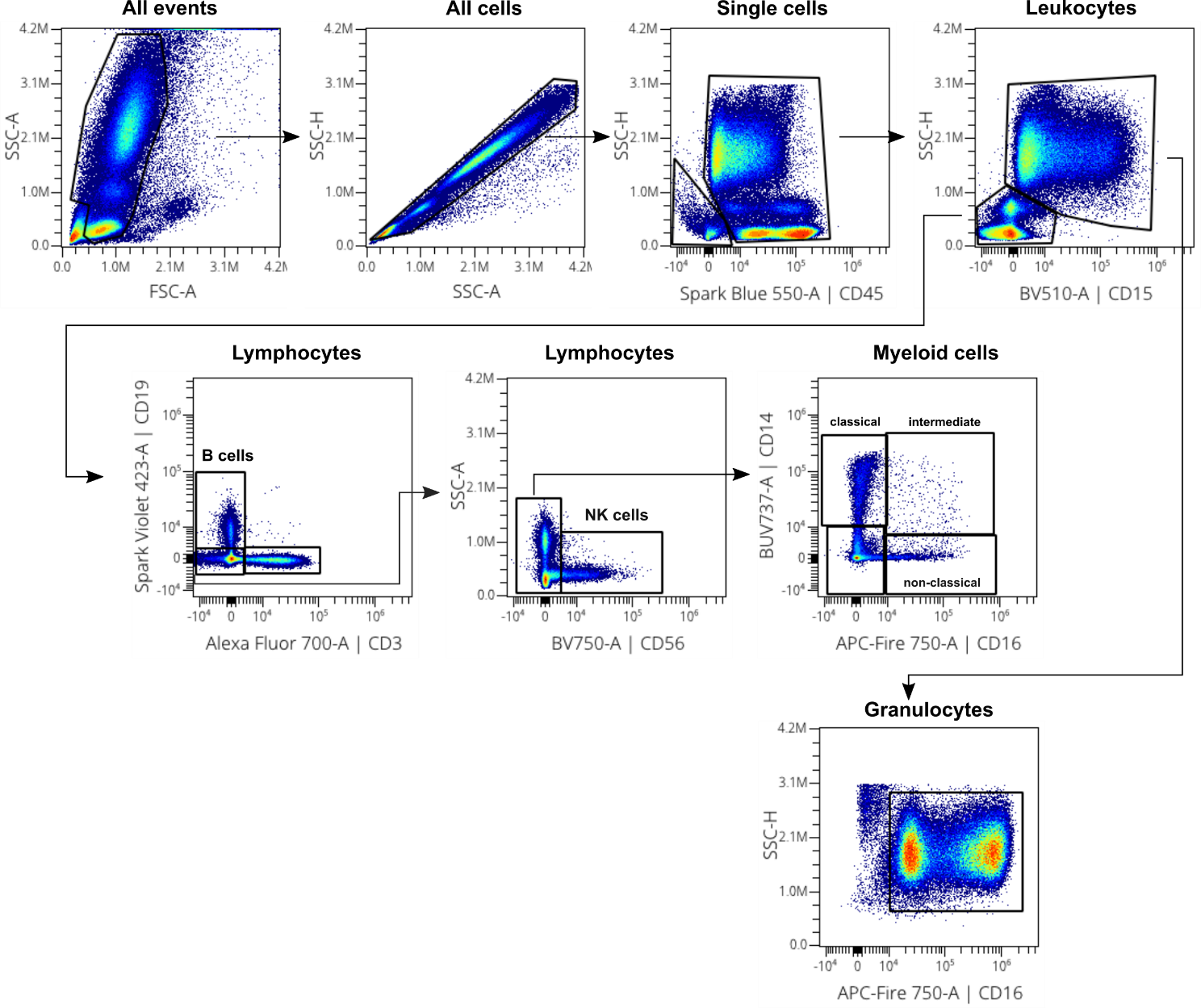


**Representative flow cytometry gating strategy for whole blood.** Single cells from all events were identified based on side scatter-area (SSC-A) and forward scatter-area (FSC-A) properties and exclusion of doublets. Leukocytes were gated based on CD45+ events, which were further distinguished into CD15+ and CD15- populations. Neutrophils were identified as CD16+ events on CD15+ populations. B cells were identified as CD19+ and CD3- on CD15- cells. Within CD3- and CD19- cells, NK cells were identified as CD56+ cells. From the CD56- population, monocytes were assessed for CD16 and CD14 expression, allowing for further categorisation into classical monocytes (CD14+ CD16-), intermediate monocytes (CD14+ CD16+) and non-classical monocytes (or macrophages) (CD14- CD16+).

**Supplementary Figure 2.**

**(a)**


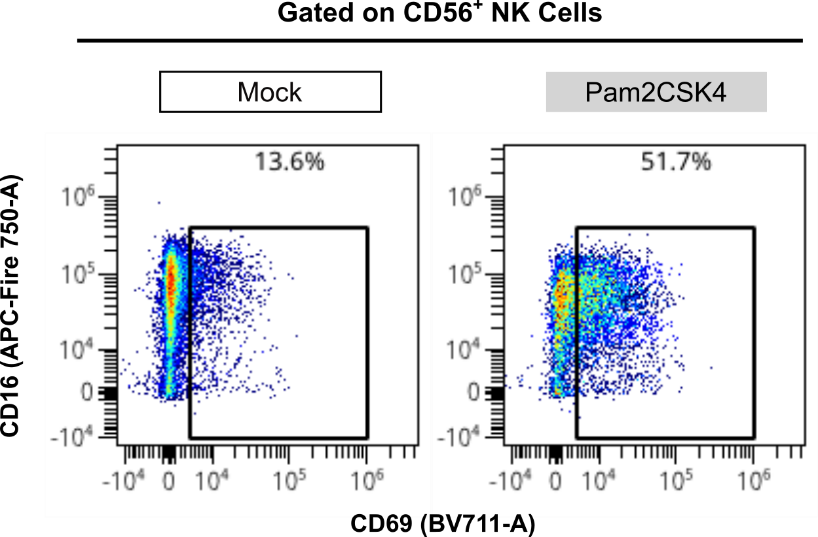


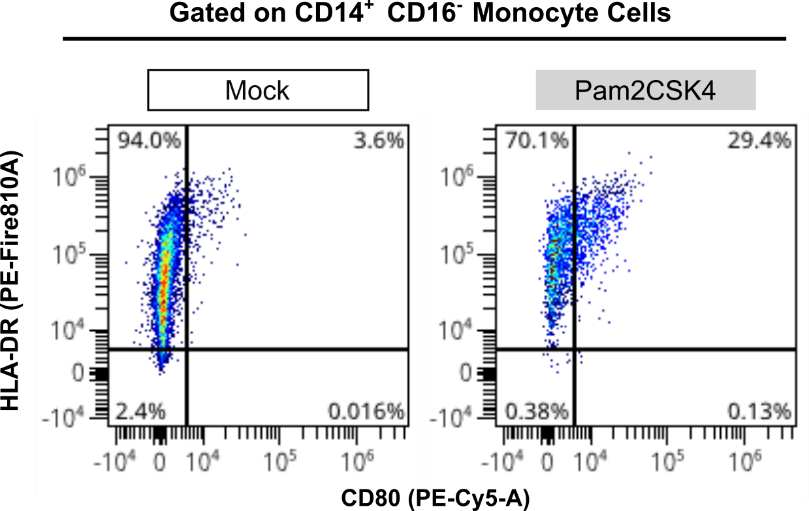


**(b)**

**(c)**


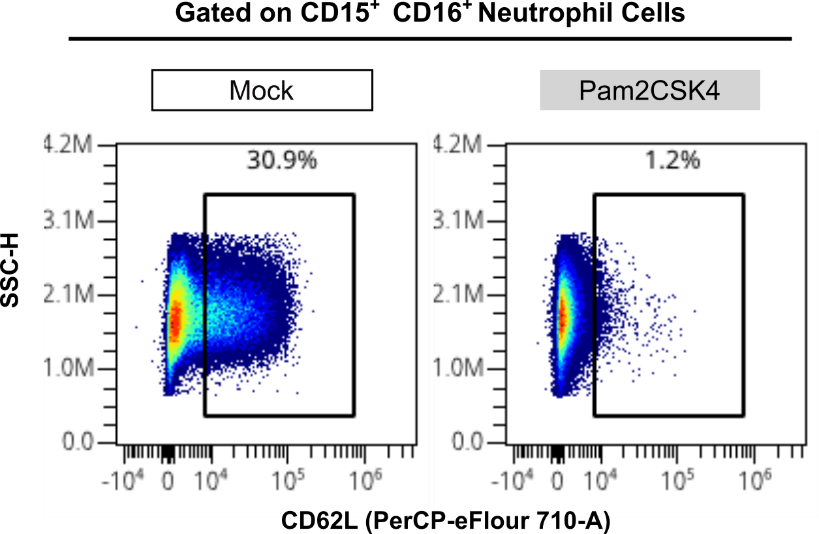


**Flow cytometry plots illustrating expression of classical activation markers on various immune subsets in whole human blood.** Whole human blood cultures were either stimulated with Pam2CSK4 ligand (10 µM) or buffer (mock) for 6 hours at room temperature and subsequent cultures were acquired on the Cytek® Aurora. Shown are flow cytometry plots demonstrating expression of CD80 on CD56+ natural killer cells (a), CD80 on CD14+, CD16- classical monocytes (b) and CD62L on CD15+, CD16- neutrophils (c).


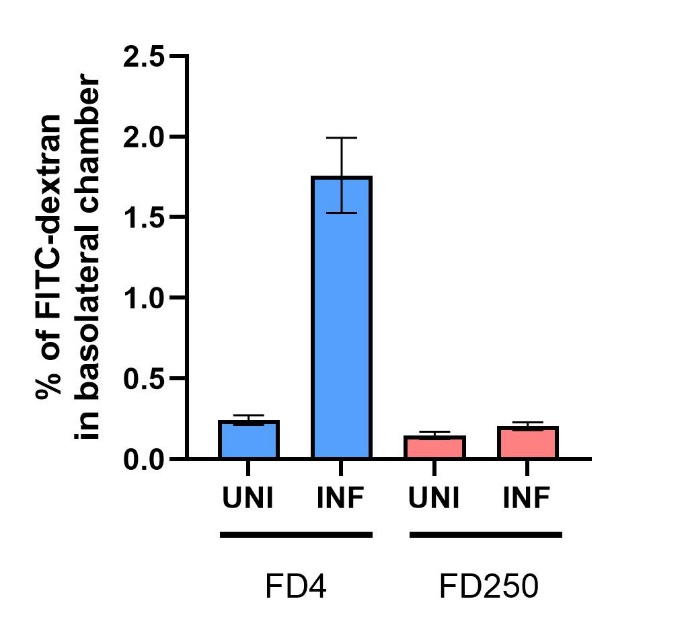

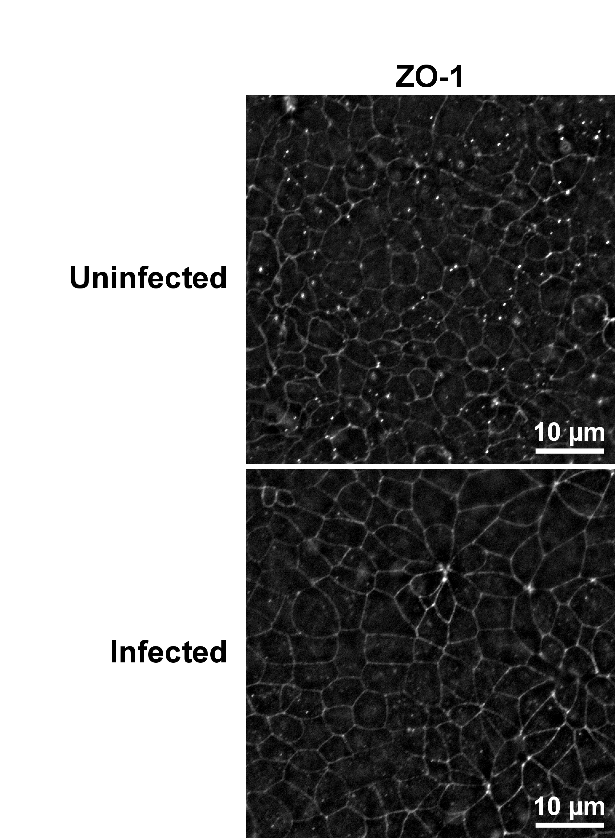
**Supplementary Figure 3.**

**(a)**

**(b)**

**Junctional integrity of Transwell grown Caco-2 cells during rotavirus infection.** Caco-2 cells were seeded on semi-permeable transwell inserts with a 0.4 µm pore size and differentiated. At 16 hpi the paracellular permeability to FD4 and FD250 from the apical to the basolateral compartment was determined (a). The formation of tight junctions was assessed by immunofluorescence staining of ZO-1 in transwell grown Caco-2 cells at 16 hpi under mock or rotavirus infection conditions (b). Images were acquired on a Nikon Ti-e using an Andor Clara camera and compiled using Nikon Elements software. Scale bar, 10 µM.
